# SEC61G regulates breast cancer cell proliferation and metastasis by affecting the Epithelial-Mesenchymal Transition

**DOI:** 10.7150/jca.65879

**Published:** 2022-01-01

**Authors:** Lingli Jin, Danxiang Chen, Suzita Hirachan, Adheesh Bhandari, Qidi Huang

**Affiliations:** 1 Department of Breast Surgery, The First Affiliated Hospital of Wenzhou Medical University, Wenzhou, Zhejiang, PR China, 325000.; 2 Department of Surgery, Breast and Thyroid Unit, Primera Hospital, Kathmandu, Nepal.; 3 Department of Surgery, Breast Unit, Tribhuvan University Teaching Hospital, Kathmandu, Nepal.

**Keywords:** breast cancer, SEC61G, proliferation, metastasis, EMT, nomogram, immune infiltration

## Abstract

Breast cancer is a common malignant tumor for women and its incidence has increased constantly in recent decades. The underlying molecular means of breast tumorigenesis endure uncertain. With the sequencing expertise, we found that the SEC61G gene is overexpressed in tumor tissues. However, the biological function of SEC61G in breast malignancy has yet to be determined.

We investigated the SEC61G expression level, genetic alteration, IHC, immune infiltration, diagnostic value, survival analysis, and functional enrichment analysis by bioinformatics analysis. Then, *vitro* experiments were done. We investigated that SEC61G was greater in breast cancer tissues related to adjacent non-tumor tissues through qRT-PCR. We performed proliferation, colony formation, migration, invasion assays, and EMT-related phenotype to determine the specific biological functions of SEC61G in breast cancer cell lines (MDA-MB-231, BT-549) transfected with small interfering RNA.

SEC61G expression and exon expression were higher in the tumor while the level of SEC61G methylation was higher in normal tissues. The expression level of SEC61G was connected with immune infiltration and survival and was an effective diagnostic and prognostic indicator. The functional enrichment analysis of SEC61G prompted that SEC61G might play a tumor-promoting role via the EMT pathway. *In vitro* experiments indicated that knocking down SEC61G considerably impaired the colony formation, cck-8, migration, and invasion, and induced apoptosis of the breast cancer cell lines. The *vitro* experiments also indicated that ectopic expression of SEC61G could influence EMT.

This study revealed that SEC61G plays vital tumorigenic functions and acts as a novel oncogene in breast cancer.

## Introduction

Breast cancer is one of the well-known malignancy cancer in women with 268,670 newly estimated diagnosed cases and 41,400 estimated deaths in the United States in 2018 1. Several types of research revealed that unlimited growth and metastasis are the leading causes of these deaths 2, 3. Remarkable progress has been made in recent years and breast cancer can be treated with endocrine, surgery, targeted or cytotoxic therapies. However, the therapeutic effect is not satisfactory 4-6. With the development of technology, hundreds of oncogenes involved in breast cancer have been discovered, and they could change from normal cells to cancer cells 7-10. Despite this outcome, the underlying mechanism of breast cancer remains many unknown mysteries.

Epithelial-mesenchymal transition (EMT) is a physiological method in which epithelial cells lose the ability of cell polarity and gain the skill of metastasis to become mesenchymal cells 11-13. A growing number of articles demonstrate that EMT is intently linked to the development of cancer 14, 15. Macrophages and fibroblasts could contribute to tumorigenesis through induction of EMT 16. Tingli Sun et al proved that miR-15a could mediate the EMT in renal tubular epithelial cells which are promoted by high glucose 17. MiR-200 family has a negative correlation with ZEB which could regulate EMT. Adam L et al. reported that miR-200 could regulate EMT in bladder cancer cells and reverses resistance to EGFR therapy 18.

To search for some novel biomarkers in breast cancer, we achieved RNA sequencing of twenty-three pairs of breast cancer and adjacent normal tissues in our unpublished report. In the result, we found SEC61 translocon gamma subunit (SEC61G) is remarkably upregulated in breast cancer related with adjacent non-cancerous tissues. Then we evaluated the RNA-seq data of breast tumor from TCGA and found the SEC61G is higher in breast cancer associated with the normal tissues too. So, we selected the SEC61G for further analysis. The SEC61 complex is the central component of the protein translocation apparatus of the endoplasmic reticulum (ER) membrane. SEC61G is one of the units that make up the SEC61 complex. Servidei T et al found a novel SEC61G-EGFR fusion gene in pediatric ependymomas through expanding stem cells without exogenous mitogens 19. Nevertheless, the potential part of SEC61G in cancer development, specifically in proliferation and metastasis, remains unclear.

In our study, first of all, we investigated the SEC61G expression level, genetic alteration, IHC, immune infiltration, diagnostic value, survival analysis, and functional enrichment analysis by bioinformatics analysis. These results remind its possible tumorigenic role in breast cancer. To further validate our guess, *vitro* experiments were done. We investigated that SEC61G was higher in breast cancer tissues associated to adjacent non-tumor tissues through qRT-PCR. We also studied the purpose of SEC61G in breast cancer cell lines via small interfering RNA (si-RNA), as well as analyzed the association amongst SEC61G expression and clinical features. The present study targeted to demonstrate the function of the SEC61G gene in breast cancer.

## Materials and Methods

### Patients and breast tissue samples

In this study, we gained 35 pairs of breast cancer tissues and paired adjacent normal tissues from the Department of Thyroid & Breast Surgery, The First Affiliated Hospital of Wenzhou Medical University between 2016 and 2017. All of the patient-derived specimens were collected and recorded with the protocols which were permitted by the ethical standards of the Ethics Committee of the First Affiliated Hospital of Wenzhou Medical University (Approval number: 2012-57). 35 pairs of fresh breast cancer tissues and normal tissues were snap-frozen in liquid nitrogen and stored at -80 °C immediately. The mRNA expression data of breast cancer were downloaded from the TCGA data portal (https://tcga-data.nci.nih.gov/tcga/). Gene expression data were accessible for 1099 breast cancer samples associated to 113 normal samples.

The primary case inclusion criteria were as follows: (1) patients whose primary tumor was breast cancer with no other organs severely affected; (2) patients who had undergone breast conservation surgery or radical mastectomy and had not received chemotherapy or radiotherapy; (3) patients with no medical history of other malignant tumor; and (4) patients with complete clinical baseline characteristics. The primary case exclusion criteria were as follows: (1) patients with other malignancies or a history of other malignancies; (2) patients suffering from serious illness such as heart failure, stroke or chronic renal failure.

### Characteristics analysis

The normalized RNA-seq data and corresponding clinical evidence were obtained from The Cancer Genome Atlas (TCGA) genomic dataset (https://cancergenome.nih.gov/). The format of RNAseq was transformed from Fragments Per Kilobase per Million (FPKM) to transcripts per million reads (TPM) and done the log2 conversion. The clinicopathologic factors of the validated cohort were collected from The First Affiliated Hospital of Wenzhou Medical University. The SEC61G high expression set and the low expression set were distinguished by the median of whole TCGA or validated cohort breast cancer samples' SEC61G expression level. We evaluated the association between SEC61G expression and clinicopathologic factors by the based package of R software (3.6.3 Version).

### Gene expression analysis

The subtypes of breast cancer in the TCGA database which had no enough normal tissues to make statistics analysis were supplemented corresponding normal tissues of the Genotype-Tissue Expression (GTEx) database by “Expression analysis -Box Plots” module of the Gene Expression Profiling Interactive Analysis, Version 2 (GEPIA2, http://gepia.cancer-pku.cn/). The visualization of SEC61G expression in different clinical subgroups was implemented by the ggplot2 package (3.3.3 Version) of R software (3.6.3 Version). GraphPad Prism software (7.0 Version) was used for analyzing the different expressions of the validated cohort and TCGA cohort.

### ROC curve and survival analysis

PROC package (1.17.0.1 Version) and ggplot2 package (3.3.3 Version) of R software (3.6.3 Version) were used to draw the ROC curve. We obtained survival maps and survival plots from GEPIA2. And statistical analysis of the association between SEC61G and survival was examined by COX regression model using survival package (3.2-10 Version) of R software (3.6.3 Version).

### Nomogram and prognostic model

We constructed a nomogram to predict the probability that one breast cancer patient can live one year, 3 years, and 5 years based on multivariate Cox regression analysis. RMS package (6.2-0 Version) and survival package (3.2-10 Version) of R software (3.6.3 Version) were performed as tools that draw this nomogram. C-index was the evaluation index that judged the nomogram's concordance.

### Immune infiltration analysis

GSVA package (1.34.0 Version) of R software (3.6.3 Version) was used for immune infiltration analysis. The algorithm used was the ssGSEA (single-sample Gene Set Enrichment Analysis) method. Spearman correlation coefficients were used to evaluate the connection between SEC61G expression and immune infiltration. And the information of the Stromal score and ESTIMATE score corresponding to the TCGA samples were downloaded from the ESTIMATE web (https://bioinformatics.mdanderson.org/estimate/).

### The genetic alteration analysis

The alteration characteristics of SEC61G which contained mutation type, alteration frequency, and copy number alteration were analyzed by UCSC Xena (https://xenabrowser.net/) and cBioPortal (https://www.cbioportal.org). The data of cBioPortal and UCSC Xena were from the TCGA database.

### SEC61G-binding proteins and SEC61G-correlated genes analysis

The top 50 SEC61G-binding proteins supported by experimental evidence were downloaded by using the STRING (https://string-db.org/) tool and the top 100 SEC61G-correlated genes were downloaded from GEPIA2. The heatmap of the top 100 SEC61G-correlated genes was drawn by Tumor Immune Estimation Resource 2 version (TIMER2, http://timer.cistrome.org/) and ggplot2 package (3.3.3 Version) of R software (3.6.3 Version). The scatter diagrams of the top 5 SEC61G-correlated genes and the Venn diagram were also drawn by the ggplot2 package (3.3.3 Version) of R software (3.6.3 Version).

### Functional enrichment analysis

Gene ontology (GO) enrichment analysis and Kyoto encyclopedia of genes and genomes (KEGG) pathway analysis were achieved by cluster profile package (3.14.3 Version) and org.Hs.eg.db package (3.10.0 Version) of R software (3.6.3 Version). The data of Gene Set Enrichment Analysis (GSEA) were from MSigDB Collections (https://www.gsea-msigdb.org/gsea/msigdb/index.jsp) and GSEA was performed by ggplot2 package (3.3.3 Version) of R software (3.6.3 Version). Protein-protein interaction (PPI) network analysis was achieved by STRING (https://string-db.org/).

### Cell cultures and growth conditions

MDA-MB-231, BT-549, SK-BR-3, MDA-MB-468, MCF-7, MDA-MB-453, BT-474, ZR-75-1, MDA-MB-436, T-47D, HS578T, and MCF-10A cells were used in this study. All cells were obtained from Shanghai Cell Biology, Institute of the Chinese Academy of Sciences (Shanghai, China). MDA-MB-231, MCF-7, BT-474, ZR-75-1, T-47D, HS578T, and SK-BR-3 were cultured in Dulbecco's Modified Eagle's Medium DMEM(Gibco, Grand Island, NY, USA) with 10% FBS (Gibco, Grand Island, NY, USA). BT-549 were cultured in RPMI-1640 medium (Gibco, Grand Island, NY, USA) supplemented with 10% FBS (Gibco, Grand Island, NY, USA). MDA-MB-468, MDA-MB-436, MDA-MB-453 were cultured in L-15 medium (Gibco, Grand Island, NY, USA) with 10% FBS (Gibco, Grand Island, NY, USA). MCF-10A cells were cultured in DMEM-F12 (Gibco, Grand Island, NY, USA) supplemented with 10% FBS (Gibco, Grand Island, NY, USA). MDA-MB-468, MDA-MB-436, and MDA-MB-453 cells were incubated in a standard cell culture incubator (Thermo, Waltham, MA, USA) at 37℃ without CO2 while the others were incubated in a standard cell culture incubator (Thermo, Waltham, MA, USA) at 37 ℃ with 5% CO2.

### Cell transfection

MDA-MB-231 and BT-549 (120,000 cells) were transfected with siRNA and Lipofectamine RNAiMAX transfection reagent (Invitrogen). Breast cancer cell lines were plated in 6-well plates 24h before cell transfection. SEC61G was silenced with 10nM siRNA for 48h. The siRNA sequences used in the study are: SEC61G siRNAs target the following sequences: SEC61G siRNA-1, Forward 5′- GCAGUUUGUUGAGCCAAGUTT -3′and Reverse 5′- ACUUGGCUCAACAAACUGCTT -3′; SEC61G siRNA-2, Forward 5′- GGGAUUCAUUGGCUUCUUUTT -3′ and Reverse 5′- AAAGAAGCCAAUGAAUCCCTT -3′; Both siRNAs were provided by Genepharma.

### RNA extraction and real-time quantitative polymerase chain reaction (RT-qPCR)

According to the manufacturer's guidelines (Invitrogen, USA), RNA from cells was isolated by TRIZOL reagent (Invitrogen, USA). The purity of RNA was inspected at 260/280nm by spectrophotometry (Thermo, San Jose, CA, USA). All RNA samples were reversed transcription (Toyobo, Osaka, Japan). Real-time reactions were run and analyzed by a Real-Time PCR system (Applied Biosystems 7500). The expression level of GAPDH mRNA was used for the normalization. The sequences of the primers used were:

SEC61G Forward: 5′-ATGGCAACAGCAATAGGAT-3′ and Reverse: 5′-ACACTTGTTCACCAATCTCT-3′; GADPH Forward: 5'-GTCTCCTCTGACTTCAACAGCG-3' and Reverse: 5'-ACCACCCTGTTGCTGTAGCCAA-3'.

### Migration and Invasion assay

The metastasis capabilities of breast cancer cells were examined through 24-well plates with 8-μm pore size inserts (3422, Corning). For migration and invasion assays, cells were collected in the medium which supplements with 10% FBS. The cells (migration assays: 4×10^4^ for MDA-MB-231 and BT-549; invasion assays: 5×10^4^ for MDA-MB-231 and BT-549) were cultured into the upper chamber. 600 µl medium containing 20% FBS was filled in the lower chamber. After 20-24 h, fixing the membrane with 4% paraformaldehyde and stained with 0.4% crystal violet solution for 10 min. Cell migration or invasion ability was estimated by the number of cells that had migrated or invaded through the membrane. Each assay was repeated three times. Selecting five random fields of view and capturing images under a microscope at a magnification of ×20.

### Cell proliferation assay

For the colony-forming assay, placed MDA-MB-231 and BT-549 cells (2×10^3^ cells) in 6-well plates and maintained in 10% FBS for 6-8 days. Then fixed plates with 4% paraformaldehyde and stained with 0.4% crystal violet solution for 10 min. These images were captured by the camera. Using the CCK-8 assay to assess the cell proliferation, MDA-MB-231 and BT-549 cells (2×10^3^ cells) were plated into 96-well plates 24h and then transfected with siRNA or siNC. The cell proliferation was determined every 24h following the manufacturer's protocol. Each assay was repeated three times.

### Apoptosis Detection Analysis

Annexin-V-FITC apoptosis detection kit was purchased to detect apoptosis. Centrifuging cells at 1000 rpm for 3 min three times and resuspending it with PBS. Discarding the supernatant, resuspending cells in 500 μL binding buffer, and mixing with 5 μL Annexin V-FITC (Kaijibio, Nanjing, China). The data was examined by flow cytometer (Guava easyCyte HT, Millipore, USA).

### Western blot analysis

Proteins were extracted by a lysis buffer and quantified with bicinchoninic acid (BCA) protein quantification kit (KeyGen Biotech). Then proteins were separated by SDS-polyacrylamide gel electrophoresis (BioRad, Berkeley, CA, USA) and transferred to polyvinylidene fluoride membranes (Millipore). Primary antibodies were as follows: N-cadherin, Vimentin, E-cadherin, and human β-actin (all Cell Signaling Technology, Danvers, MA, USA).

### Statistical Analysis

All assays were achieved in triplicate. The Shapiro-Wilk test was used to test the distribution of data. If variables obeyed normal distribution, they would be investigated by Student's t-test. If variables did not obey normal distribution, they would be analyzed by the Wilcoxon test or Mann Whitney test. Two group comparisons were assessed by Student's t-test, and one‑way ANOVA was used to analyze multiple group comparisons. Spearman correlation analysis was used to assesses the monotonic relationships between two continuous or sequential variables. A log-rank test was used to analyze the difference between the two groups' Kaplan-Meier survival curves.

Chi-square test and logistic regression were used for analyzing the correlation between SEC61G expression and clinicopathological features. Cox regression was used to analyze the animation prognosis of breast cancer patients. The statistical analyses were performed using SPSS 25.0 software, GraphPad Prism software (7.0 Version), and R 3.6.3 software. The differences were considered to be statistically significant at P < 0.05. The data visualization was accomplished by R 3.6.3 software and GraphPad Prism software (7.0 Version).

## Result

### SEC61G is overexpression in breast cancer

**Figure 1:**
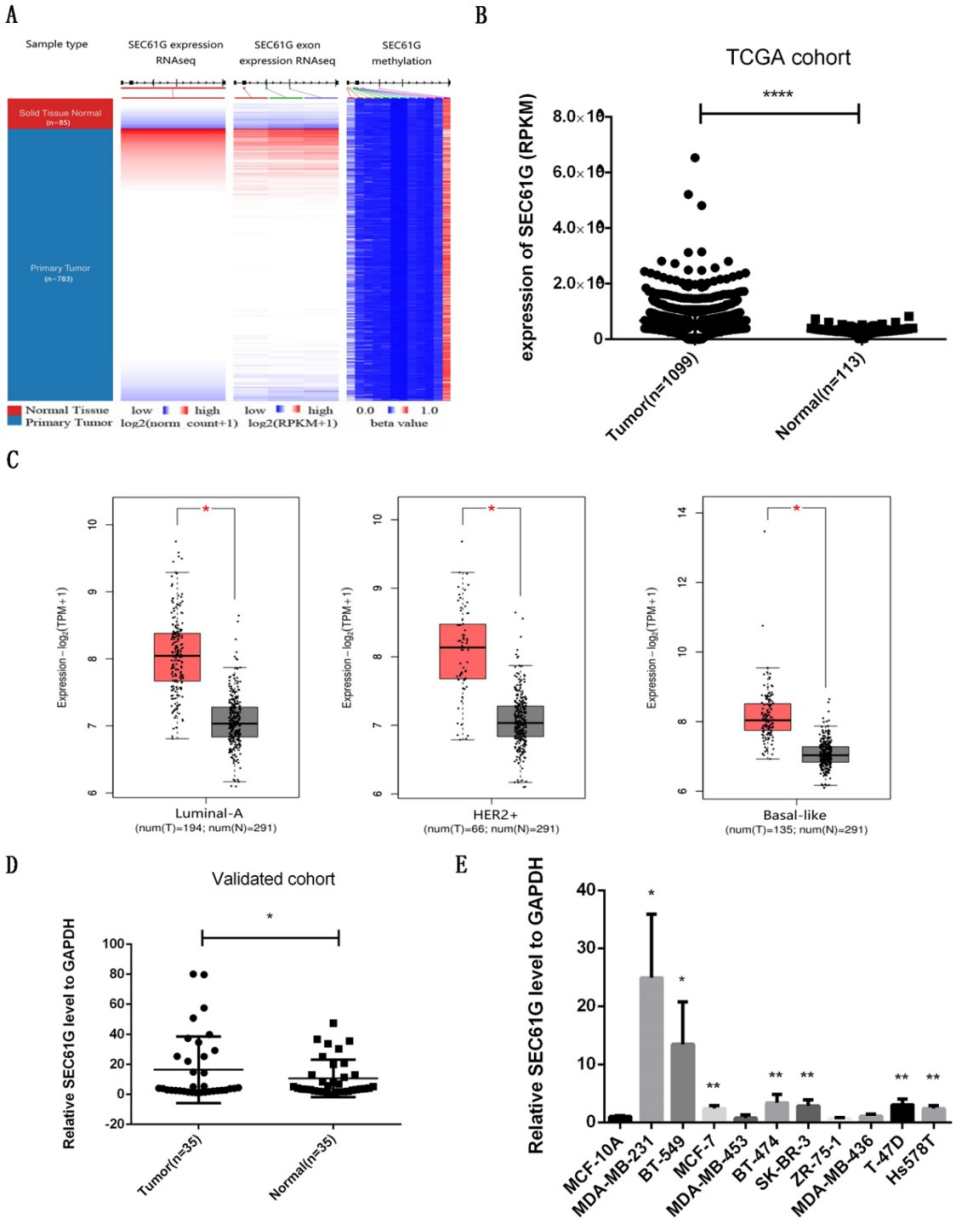
The expression level of SEC61G in breast cancer. (A) The heat maps of SEC61G mRNA expression, exon expression, and methylation in primary breast cancer and corresponding normal tissues. (B) The SEC61G expression level of different cancers in the TCGA dataset. (C) SEC61G expression of different breast cancer subtypes in TCGA+GTEx dataset. (D) SEC61G expression in breast cancer invalidated cohort. (E) SEC61G expression in different breast cell lines. * P<0.05, **p<0.01, ***P<0.001, **** P <0.0001.

**Figure 2:**
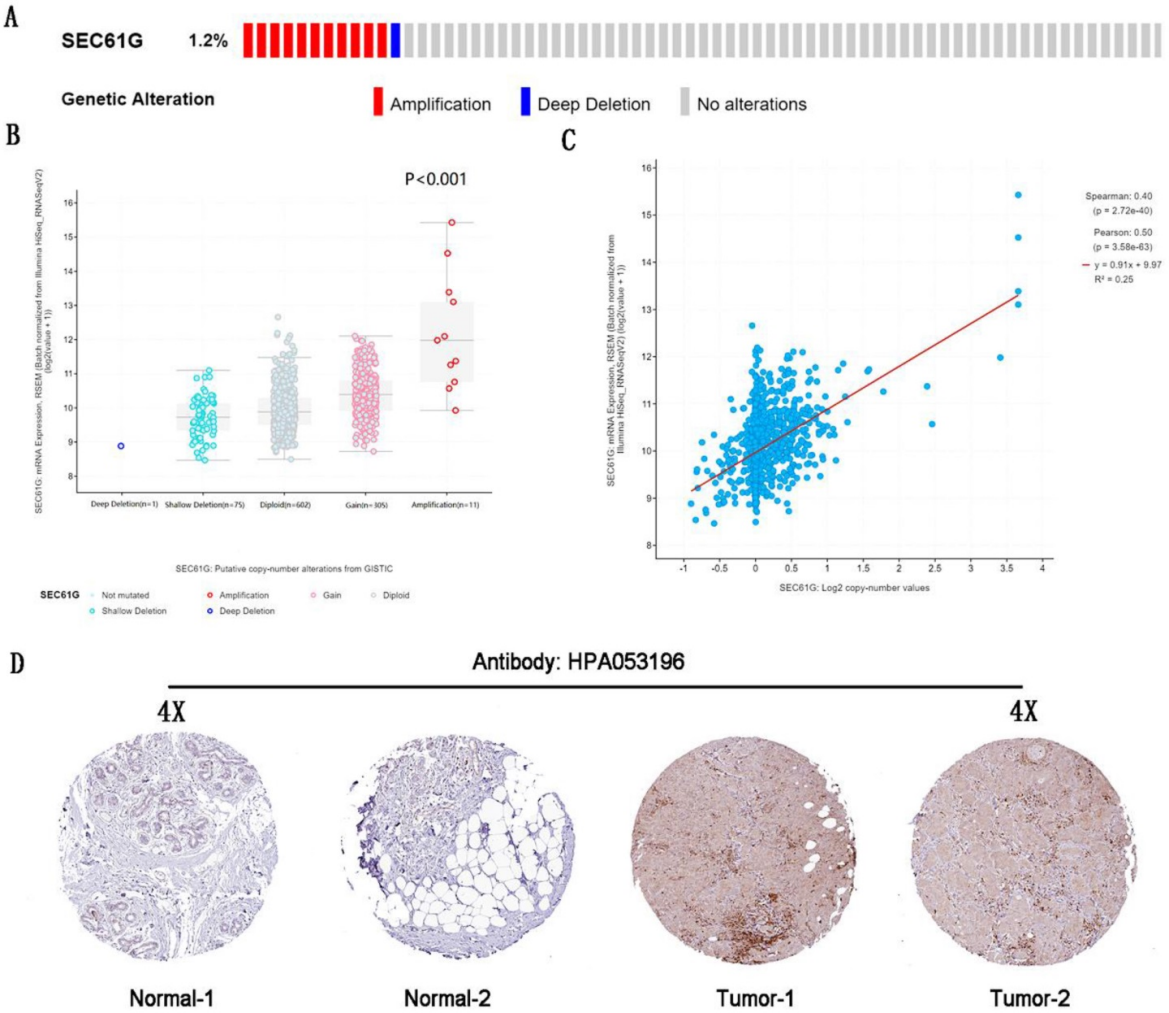
The genetic alteration and IHC of SEC61G. (A) The genetic alteration of SEC61G in breast cancer. (B) The correlations between the mRNA expression level and genetic alterations of SEC61G. (C) The correlations between the mRNA expression level and copy number of SEC61G. (D) IHC slices of SEC61G in the breast. * P<0.05, ** P <0.01, *** P <0.001, **** P <0.0001.

To validate the consequence of RNA sequencing, we assessed the level of SEC61G expression, exon expression, and methylation in the TCGA cohort. As shown in Figure 1A, we discovered that SEC61G expression and exon expression were higher in the tumor while the level of SEC61G methylation was higher in normal tissues. The statistical analysis about SEC61G level in TCGA, our local validated cohort, and different breast cell lines also conformed to this result (Figure 1B-E). The immunohistochemistry (IHC) from the Human Protein Atlas database also verified that the SEC61G protein was over-expressive in breast tumors (Figure 2D). The basic information about these two IHC slices was listed in Table S1. These results showed that SEC61G was overexpressed in breast cancer and might function as an oncogene in breast cancer.

### The genetic alteration of SEC61G in breast cancer

By analyzing the molecular characteristics of SEC61G, we found that SEC61G was altered by 1.2% in the tested breast cancer samples. And amplification was the most common alteration (Figure 2A). And the analysis about the correlations between the mRNA expression level, genetic alterations, and copy number of SEC61G was done to conform amplification would increase the mRNA expression level (Figure 2A-C). These further validated that SEC61G was overexpressed in breast cancer and may function as an oncogene in breast cancer.

### Correlations between the expression of SEC61G and immune infiltration

**Figure 3:**
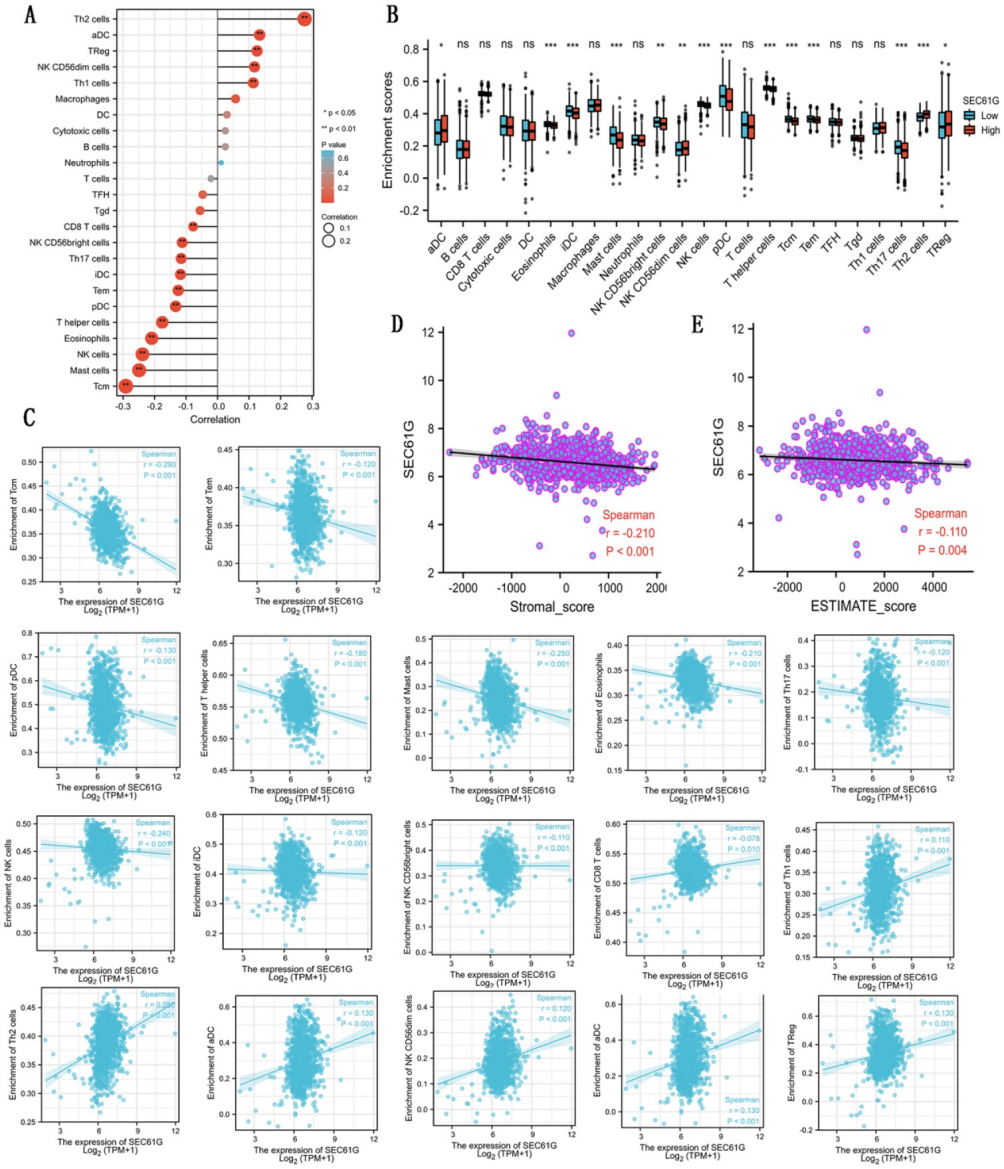
Correlations between the expression level of SEC61G and immune infiltration. (A) The lollipop figure about the correlations between the expression level of SEC61G and immune infiltration of different immune infiltration cells. (B) The bar graph about the immune infiltration level of different immune infiltration cells in SEC61 high set and low set. (C) The scatter diagrams about the correlations between the expression level of SEC61G and the immune infiltration level of different immune infiltration cells. (D)-(E) The scatter diagrams about the correlations between the expression level of SEC61G and stromal score (D) and ESTIMATE score (E). * P<0.05, ** P <0.01, *** P <0.001, **** P <0.0001.

As shown in Table 5 and Figure 3, in different immune cell types, CD8 T cells, NK D56bright cells, Th17 cells, iDC, Tem, pDC, T helper cells, eosinophils, NK cells, mast cells, and Tcm were negatively connected with the expression level of SEC61G. And Th2 cells, aDC, Treg, NK CD56dim cells, and Th1 cells were prominently positively connected with the expression level of SEC61G. At the same time, there were negative correlations between SEC61G expression level and stromal score, ESTIMATE score. These indicated that a high expression level of SEC61G might predict a poor prognosis of breast cancer. SEC61G might affect tumor immunity, which might guide the immunological therapy of breast cancer.

### Diagnostic value of the expression of SEC61G in breast cancer

**Figure 4:**
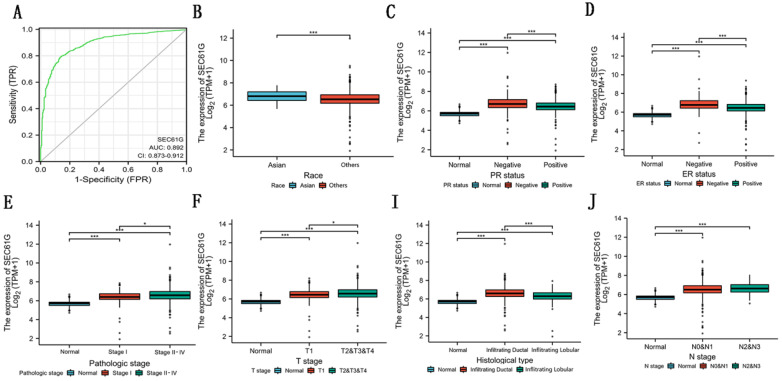
Diagnostic value of the expression of SEC61G and the correlation between SEC61 expression and clinical factors in TCGA. (A) ROC curve for differentiating normal people and breast cancer patients. (B)-(I) The correlation between SEC61 expression and race (B), PR status (C), ER status (D), pathological stage (E), T stage (F), histological type (I), and N stage (J). * P<0.05, ** P <0.01, *** P <0.001, **** P <0.0001.

In this study, to evaluate the diagnostic value of SEC61G expression in breast cancer, ROC curve analysis was conducted. As showed in Figure 4A, the Area Under the Curve (AUC) of SEC61G was 0.892, which indicated a high diagnostic value in differentiating tumor and normal.

### The relationship between SEC61G expression and clinical features

To know the relation among SEC61G and breast cancer clearly, we examined the relationship of SEC61G with clinicopathologic qualities. We divided the patients into high-expression groups and low-expression groups according to the median value in the TCGA cohort. The results discovered that race (P=0.006), histological type (P<0.001), PR status (P<0.001), ER status (P<0.001), tumor stage (P=0.013), lymph node metastasis stage (P=0.021), and pathologic stage (P=0.002) were significantly related to the SEC61G expression while distant metastasis (P = 0.065) and SEC61G expression were related to a certain extent (Table 2). As shown in Figure 4B-J, the relationship among SEC61G expression and different clinical features was visualized. These results implied that higher SEC61G expression might influence the abilities of proliferation and metastasis in breast cancer and was linked with unfavorable prognosis in breast cancer.

### The relationship between SEC61G expression and survival

**Figure 5:**
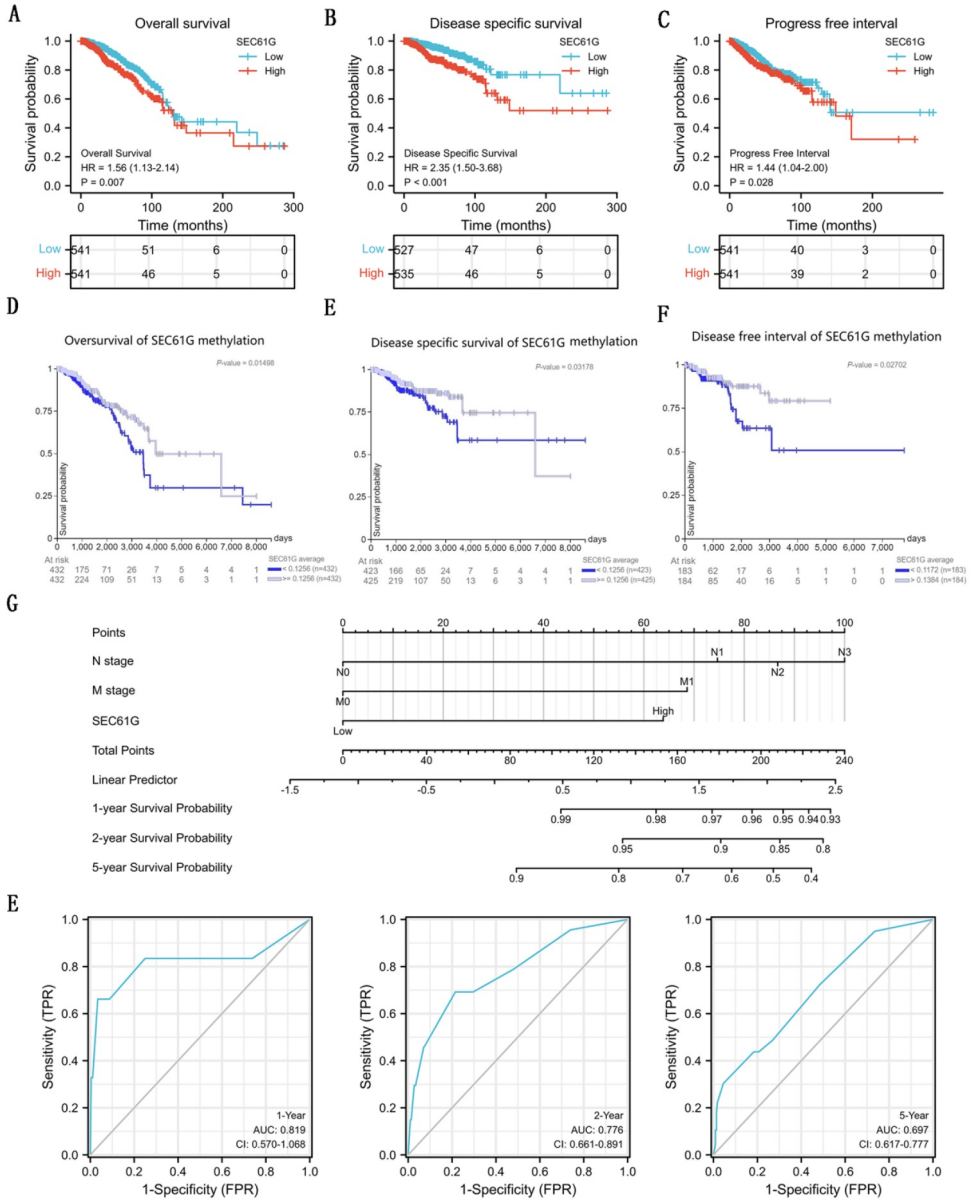
The survival analysis of SEC61G in breast cancer. (A)-(C) Overall survival (A), disease-specific survival (B), and progress-free interval (C) plots of breast cancer patients that grouped by SEC61G expression level. (D)-(F) Overall survival (D), disease-specific survival (E), and progress-free interval (F) plots of breast cancer patients that grouped by SEC61G methylation level. (G) A nomogram for predicting the 1 year, 3 years, and 5 years survival probability of breast cancer patients. (E) ROC curves of the nomogram. * P<0.05, ** P <0.01, *** P <0.001, **** P <0.0001.

To analyze the connection between SEC61G and survival, a Kaplan-Meier analysis was made. Univariate Cox regression analysis for disease-specific survival showed that higher expression of SEC61G was connected with poor disease-specific survival (P<0.001, hazard ratio [HR]=2.348, 95%CI = 1.498-3.681) (Table 3). And we found that higher disease-specific survival expression, T stage, N stage, M stage, pathologic stage, PR status, and ER status were correlated with disease-specific survival (Table 3). Then, to verify overexpression of SEC61G was an independent risk factor for disease-specific survival of breast cancer patients, multivariate analysis was made. The results of multivariate analysis for overall survival showed that high SEC61G expression was an independent risk factor for disease-specific survival of breast cancer patients (P=0.013, hazard ratio [HR]=2.164, 95%CI=1.180-3.961) (Table 4). And the survival curves of SEC61G were drawn (Figure 5A-C), which reminded us that higher expression of SEC61G predicted poor overall survival, poor disease-specific survival, and poor progress-free interval of breast cancer patients. And, survival analysis of SEC61G methylation was also showed similar consequences (Figure 5D-F). Therefore, we constructed a nomogram to predict the 1 year, 2 years, and 5 years survival probability of breast cancer patients (C-index= 0.698, 95%CI=0.670-0.726) (Figure 5G). ROC curves were conducted to evaluate the diagnostic accuracy of the nomogram. As showed in Figure 5E, the Area Under the Curve (AUC) of 1-year survival, 2-years survival, and 5-years survival respectively were 0.819, 0.776, and 0.697, which indicated the effective diagnostic value of nomogram.

### Functional enrichment analysis of SEC61G

**Figure 6:**
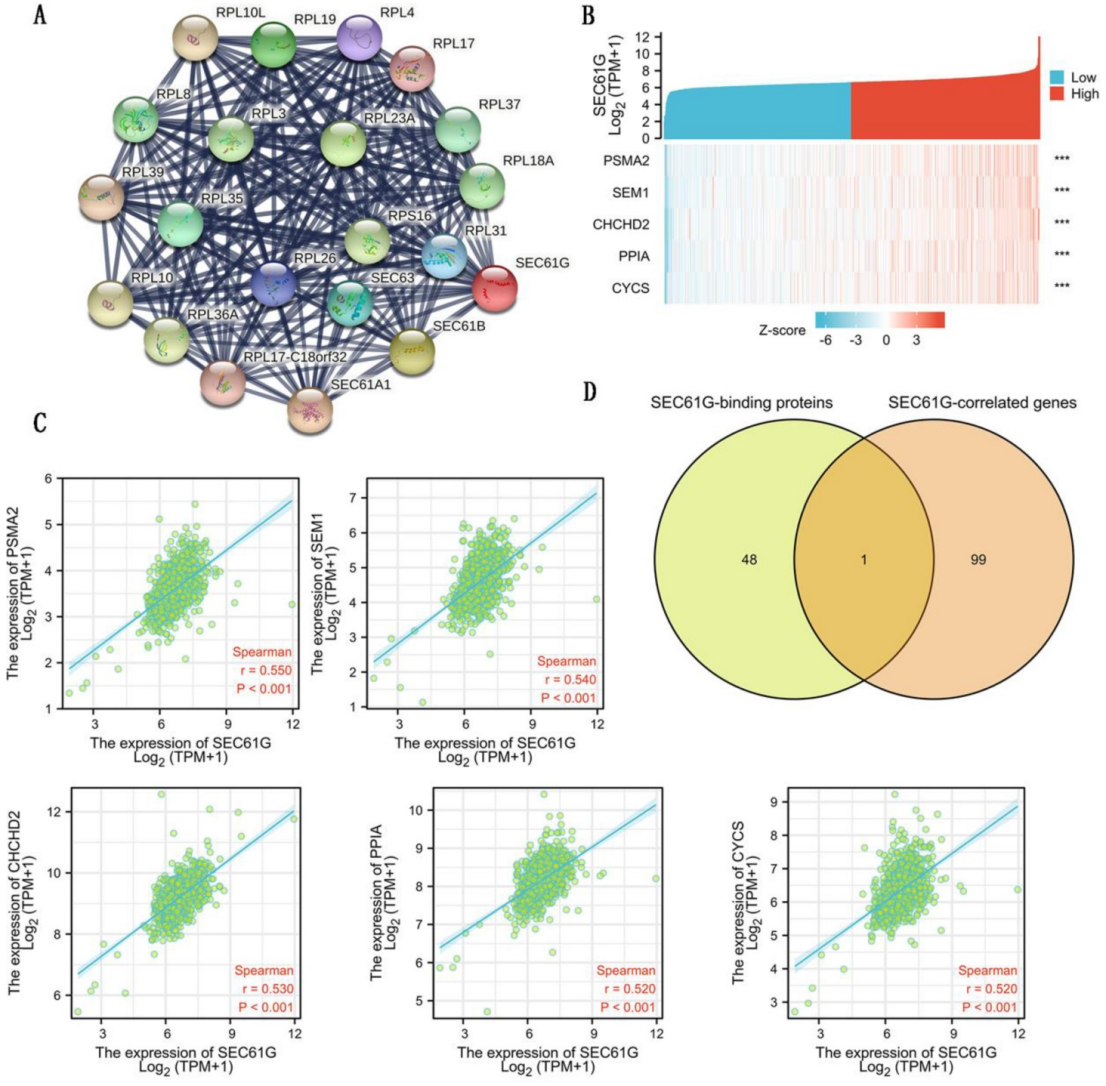
SEC61G-related gene and SEC61G-binding proteins enrichment analysis. (A) Protein-protein interaction network of SEC61G. (B) Heat map about the top 5 SEC61G-related gene enrichment. (C) The scatter diagrams about the top 5 SEC61G-related gene enrichment. (D) A Venn diagram about intersection analysis of the top 50 proteins and the top 100 genes. * P<0.05, ** P <0.01, *** P<0.001, **** P <0.0001.

**Figure 7:**
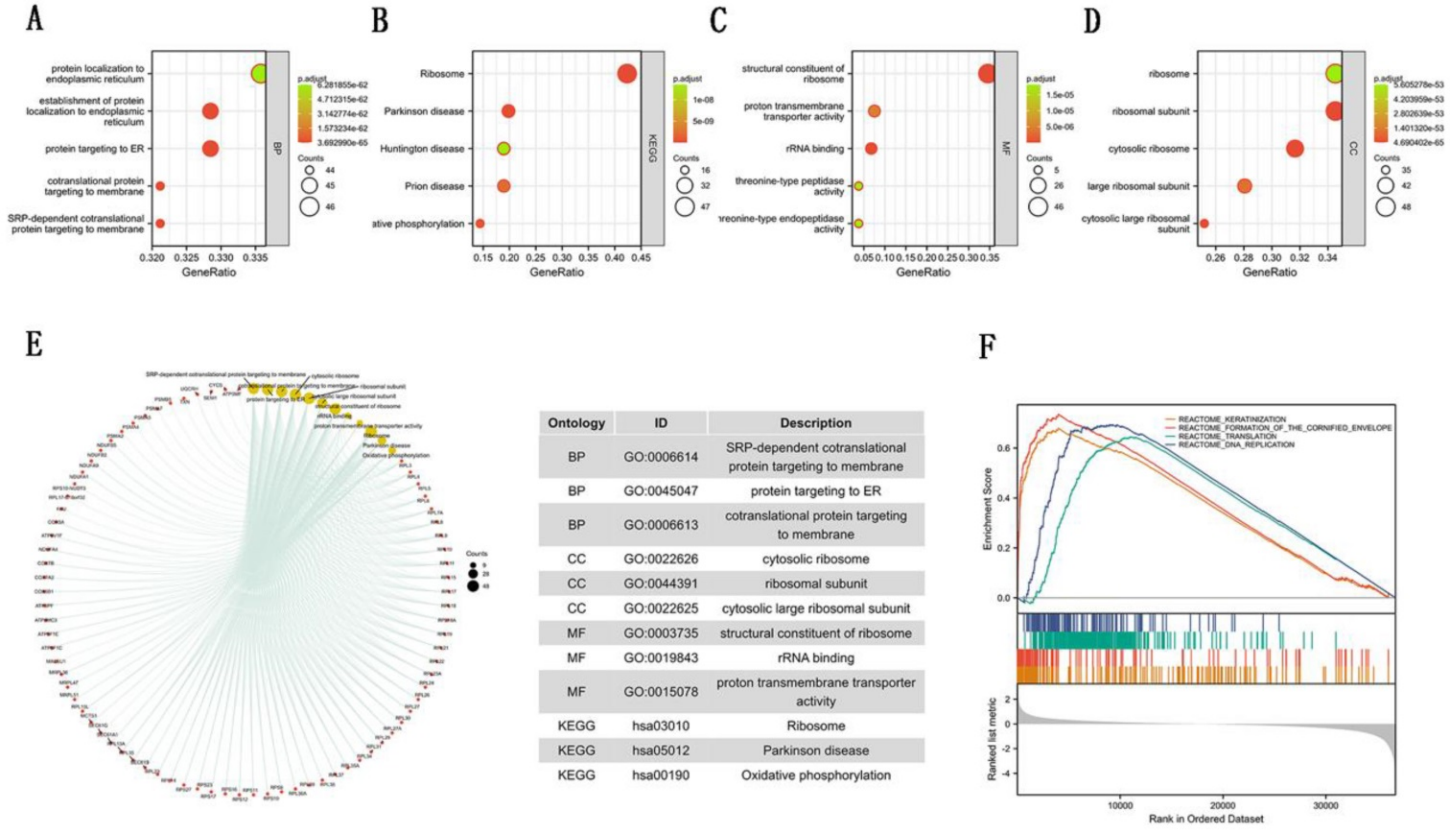
Enrichment analyses of SEC61G. (A)-(C) GO enrichment analyses of SEC61G. (D) KEGG enrichment analyses of SEC61G. (E) GO and KEGG enrichment interactive network. (F) GSEA enrichment analysis of SEC61G. * P<0.05, ** P <0.01, *** P <0.001, **** P <0.0001.

To further explore the carcinogenic mechanism of SEC61G, we downloaded the top 50 SEC61G-binding proteins supported by experimental evidence by using the STRING tool and top 100 SEC61G-correlated genes from GEPIA2. Then, pathway enrichment analyses were done. The interaction network of these to the top 20 SEC61G-binding proteins was shown in Figure 6A. As shown in Figure 6B, PSMA2 (P<0.01, R=0.55), SEM1 (P<0.01, R=0.54), CHCHD2 (P<0.01, R=0.53), PPIA (P<0.01, R=0.52) and CYCS (P<0.01, R=0.52) had the strongest SEC61G-related correlation and were significantly positively connected with the expression level of SEC61G. After an intersection analysis of the top 50 proteins and the top 100 genes, a common member called SEC61B was found (Figure 6D). Then, we combined these genes and proteins for the following GO and KEGG analyses. The GO enrichment analysis contained biological process, cellular component, and molecular function in these three major functional groups (Figure 7A-C). Figure 7D showed the KEGG analyses. Figure 7E showed the GO and KEGG enrichment interactive network, and the results revealed that SEC61G and other genes being analyzed were mainly connected with protein export, ribosome, and cadherin binding.

To identify the mainly SEC61G-related signaling pathways that are activated in breast cancer, GSEA analysis was done. And we found several significant SEC61G-related signaling pathways. In this study, we drew the top four pathways, including REACTOME_KERATINIZATION, REACTOME_FORMATION_OF_THE_CORNIFIED_ENVELOPE, REACTOME_TRANSLATION, and REACTOME_DNA_REPLICATION (Figure 7F). The Functional enrichment analysis of SEC61G prompted us that SEC61G might play a tumor-promoting role via the epithelial-mesenchymal transition (EMT) pathway.

### SEC61G knockdown effects on proliferation in breast cancer cell lines

**Figure 8:**
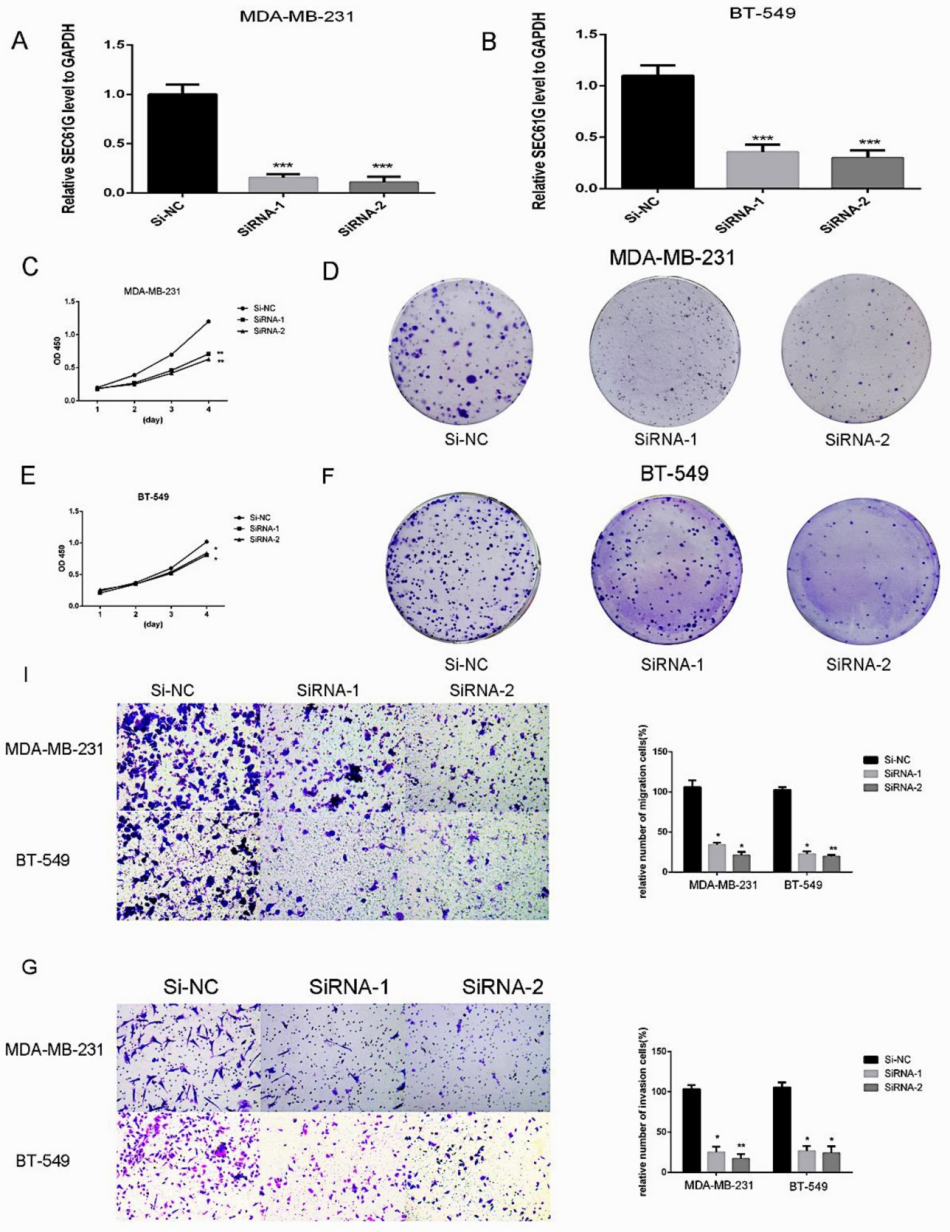
Downregulation of SEC61G gene inhibits MDA-MB-231 and BT-549 proliferation, cell colony formation, and metastasis. (A and C) Cell proliferation assay. MDA-MB-231 and BT-549 cell lines transfected with si-RNA or si-NC were cultured in 96-well plates for 1-4 days and using CCK-8 measured cell proliferation. Cell proliferation was significantly suppressed in MDA-MB-231 and BT-549 transfected by si-RNA. (B and D) MDA-MB-231 and BT-549 cell lines transfected with si-RNA or si-NC were cultured in 6-well plates for 10-14 days. (E) In MDA-MB-231 and BT-549, transwell migration assays in down-regulation SEC61G cells and their corresponding control cells. (F) In MDA-MB-231 and BT-549, transwell invasion assays in down-regulation SEC61G cells and their corresponding control cells. Quantitative results of metastasis assays. The columns represent the mean of invading cell numbers from at least three independent experiments *P < 0.05; **P < 0.01; ***P < 0.001 in comparison with the NC group using Student's t-test.

Since we found that the expression of SEC61G and tumor size were statistically significant in TCGA, we wonder if SEC61G could affect the proliferation of breast cancer cell lines. We assessed SEC61G expression level between breast cancer cell lines and normal breast cell lines through RT-qPCR. As shown in Figure 1E, SEC61G levels in the human breast cancer cell lines MDA-MB-231 and BT-549 were higher than those in the human normal breast cell line MCF-10A.So we chose MDA-MB-231 and BT-549 for the research cell lines. Then we knocked down SEC61G expression in MDA-MB-231 and BT-549 through small interfering RNA (Figure 8A-B). In Figures 8C and E, we performed CCK-8 assays to determine the role of SEC61G in breast cancer cell proliferation. Cell lines that were transfected with Si-SEC61G showed decreased proliferation capacity in CCK-8 assays. Additional cell colony formation was significantly inhibited in cells by knocking down SEC61G expression associated with control (Figure 8D and F). This finding signified that SEC61G could improve the proliferation of breast cancer cells.

### SEC61G knockdown inhibits breast cancer cells migration and invasion

Metastasis is a principal characteristic of the tumor, then we inspected the influence of SEC61G on the migration and invasion of breast cancer cells. In migration assay, MDA-MB-231 and BT-549 transfected with si-SEC61G migrated much fewer cells than the control cells (Figure 8I). As shown in Figure 8G, knocking down SEC61G might decrease the invasive capability of breast cancer cell lines. These outcomes confirmed that SEC61G as an oncogene could affect metastasis in a breast cancer cell.

### Detection of cell death mechanism

**Figure 9:**
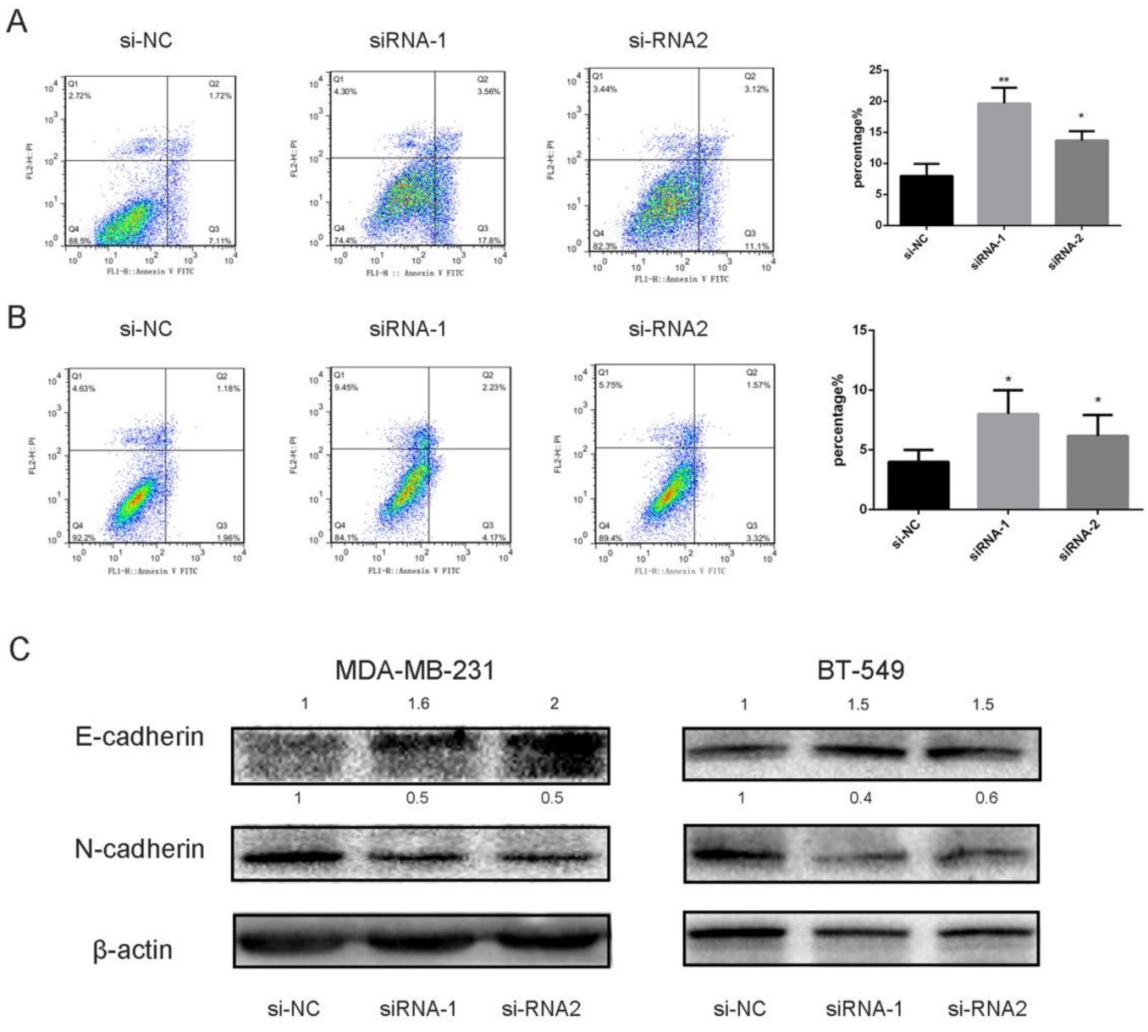
Downregulation of DEC61G increased breast cancer cell lines (MDA-MB-231 and BT-549) apoptosis via EMT. (A)-(B) Annexin V/PI assay was applied to analyze the MDA-MB-231 and BT-549 cell lines transfected with si-RNA or si-NC. Knocking down SEC61G could increase apoptotic cell death in breast cancer cells. (C) The influence of SEC61G expression on the levels of E-cadherin, N-cadherin in MDA-MB-231 and BT-549 cell lines transfected with si-RNA or si-NC by western blot. The Columns represent the mean of invading cell numbers from at least three independent experiments. *P < 0.1, **P < 0.05, ***P < 0.01 in comparison with si-NC using student's t-test.

Apoptosis is a highly controllable biologic process in which cells and other components are essentially “chopped” into pieces 20. To investigate underlying molecular mechanisms, we utilized Annexin V/PI assay to examine the cells transfected with siRNA likened with the control. Our experiment outcomes indicated that knocking down SEC61G had an impact on apoptosis in MDA-MB-231 and BT-549 (Figure 9A-B). These data presented that knockdown of SEC61G might increase apoptotic cell death in breast cancer cell lines.

### SEC61G gene promotes cancer migration and invasion by EMT

Epithelial-to-mesenchymal transition (EMT) is an important step in the morphologic transformation process and it is related to the acquisition of cancer cell metastasis in malignancies 12, 21. So, we inspected the protein expression level of epithelial markers and mesenchymal markers in breast cancer cell lines. As presented in Figure 9C, knocking down SEC61G had significantly increased E-cadherin and decreased N-cadherin expression which means SEC61G could regulate metastasis by EMT.

## Discussion

Breast cancer is the most regularly diagnosed malignancy and the key reason of cancer-related mortality in women worldwide 1, 22. In China, the incidence rate of breast cancer has ascended noticeably in recent decades 23. Estrogen receptor (ER), progesterone receptor (PR), and human epidermal growth factor receptor 2 (HER2) are shared breast cancer biomarkers, and the classification of breast cancer is mostly centered on them. It is believed that breast malignancy is a heterogeneous disease that the information provided by these parameters is always not adequate for choosing the optimal treatment 24. Though much development in breast malignancy investigation has been made, the specific molecular mechanisms of breast malignancy endure unidentified. High-throughput sequencing analysis of genetic alterations, and advanced sequencing methods, have been applied to explore its molecular mechanisms.

In our study, we assessed the level of SEC61G expression, exon expression, and methylation in the TCGA cohort, and discovered that SEC61G expression and exon expression were higher in the tumor while the level of SEC61G methylation was higher in normal tissues. The differential expression of SEC61G was further proved in the local validated cohort. The finding that amplification was the most common alteration of SEC61G in breast cancer further validated its possible tumorigenic role in breast cancer.

The immune infiltration analysis indicated that SEC61G might affect tumor immunity, which might guide the immunological therapy of breast cancer. To know more about SEC61G and breast tumor, we examined the association of SEC61G with clinicopathologic features. In TCGA cohorts, highly expression SEC61G likely induce larger tumor size (P=0.013), more lymph node metastasis (P = 0.021), and a higher risk of distant metastasis. Overall survival, disease-specific survival, and progress-free interval are worse in groups with high SEC61G expression. Based on COX regression analysis, we constructed a nomogram to predict the 1 year, 2 years, and 5 years survival probability of breast cancer patients (C-index= 0.698, 95%CI=0.670-0.726). Loss-of-function analysis indicated that SEC61G knockdown can decrease the migration and invasion of breast cancer cells *in vitro*. Moreover, down-regulated SEC61G impaired the proliferation of breast cancer cell lines which was consistent with our previous outcomes and promoted apoptotic cell death in breast tumor cells. The before functional enrichment analysis of SEC61G prompted us that SEC61G might play a tumor-promoting role via the epithelial-mesenchymal transition (EMT) pathway. So, western blotting about the EMT pathway was made. We found SEC61G downregulation could remarkably weaken N-cadherin and Vimentin expression and enhance E-cadherin expression in breast cancer cells.

Despite our outstanding findings, this research showed some limitations. On the one hand, enough quantity of the validation group should be utilized to provide reliable clinicopathological features and results. On the other hand, have yet to define the mechanism of SEC61G in breast tumorigenesis, although we demonstrated that EMT is involved in the function of this gene.

In the summary, SEC61G is one of the up-regulated genes in breast cancer. Withdrawal of SEC61G can decrease breast tumorigenesis by impairing cell colony formation and metastasis. The findings of this study offer a novel potential marker and a target in breast cancer treatment.

## Supplementary Material

Supplementary table.

## Figures and Tables

**Table 1:** Baseline characteristics of the SEC61G high expression set and the low expression set in the TCGA cohort.

Characteristic	High expression (%)	Low expression (%)	P -value
T stage (AJCC7)			0.008*
T1	154 (28.6%)	123 (22.7%)	
T2	297 (55.1%)	332 (61.4%)	
T3	77 (14.3%)	62 (11.5%)	
T4	11 (2%)	24 (4.4%)	
N stage (AJCC7)			0.037*
N0	266 (50.3%)	248 (46.4%)	
N1	184 (34.8%)	174 (32.5%)	
N2	52 (9.8%)	64 (12%)	
N3	27 (5.1%)	49 (9.2%)	
M stage (AJCC7)			0.099
M0	462 (98.7%)	440 (96.9%)	
M1	6 (1.3%)	14 (3.1%)	
Pathologic stage (AJCC7)			0.003*
Stage I	108 (20.6%)	73 (13.6%)	
Stage II	301 (57.4%)	318 (59.3%)	
Stage III	111 (21.2%)	131 (24.4%)	
Stage IV	4 (0.8%)	14 (2.6%)	
Race			< 0.001*
Asian	19 (3.9%)	41 (8.2%)	
White	411 (83.7%)	342 (68%)	
Others	61 (12.4%)	120 (23.9%)	
Age (years)			0.833
<=60	298 (55.1%)	303 (55.9%)	
>60	243 (44.9%)	239 (44.1%)	
Histological type			< 0.001*
Ductal	353 (72.3%)	419 (85.7%)	
Lobular	135 (27.7%)	70 (14.3%)	
PR status			< 0.001*
Negative	132 (25.2%)	210 (41.1%)	
Indeterminate	1 (0.2%)	3 (0.6%)	
Positive	390 (74.6%)	298 (58.3%)	
ER status			< 0.001*
Negative	79 (15.1%)	161 (31.4%)	
Indeterminate	2 (0.4%)	0 (0%)	
Positive	442 (84.5%)	351 (68.6%)	
HER2 status			0.065
Negative	295 (80.4%)	263 (73.1%)	
Indeterminate	5 (1.4%)	7 (1.9%)	
Positive	67 (18.3%)	90 (25%)	
Menopause status			0.749
Pre	115 (23.9%)	114 (23.3%)	
Peri	22 (4.6%)	18 (3.7%)	
Post	345 (71.6%)	358 (73.1%)	
Total	541	542	

Abbreviations: SEC61G, SEC61 translocon subunit gamma; AJCC7, American Joint Committee on Cancer 7th edition.

**Table 2:** The correlation between SEC61G expression and clinicopathologic factors in the TCGA cohort.

Characteristics	Total(N)	OR (95% CI)	p-value
Race (Others vs. Asian)	976	0.457 (0.256-0.789)	0.006*
Age (>60 vs. <=60)	1065	0.974 (0.765-1.240)	0.832
Histological type (Lobular vs. Ductal)	959	0.446 (0.322-0.615)	<0.001*
PR status (Positive vs. Negative)	1012	0.469 (0.358-0.612)	<0.001*
ER status (Positive vs. Negative)	1015	0.381 (0.280-0.517)	<0.001*
HER2 status (Positive vs. Negative)	705	1.423 (0.997-2.038)	0.053
Menopause status (Post vs. Pre & Peri)	956	1.118 (0.842-1.486)	0.441
T stage (T2-3 vs. T1)	1062	1.419 (1.077-1.873)	0.013*
N stage (N2&3 vs. N0&1)	1046	1.454 (1.060-2.003)	0.021*
M stage (M1 vs. M0)	909	2.480 (0.985-7.055)	0.065
Pathologic stage (Stage II-IV vs. Stage I)	1042	1.677 (1.212-2.332)	0.002*

Notes: The stages were graded according to AJCC7. Abbreviations: SEC61G, SEC61 translocon subunit gamma; AJCC7, American Joint Committee on Cancer 7th edition.

**Table 3:** Univariate COX regression analysis for disease specific survival in the TCGA cohort.

Characteristics	Total(N)	HR (95% CI)	p-value
T stage (T2-3 vs. T1)	1059	1.781 (1.033-3.071)	0.038*
N stage (N1-3 vs. N0)	1044	2.462 (1.502-4.036)	<0.001*
M stage (M1 vs. M0)	903	7.454 (3.988-13.931)	<0.001*
Pathologic stage (II-IV vs. I)	1041	3.396 (1.478-7.803)	0.004*
Age (>60 vs. <=60)	1062	0.692 (0.451-1.063)	0.093
PR (Positive vs. Negative)	1010	0.519 (0.334-0.807)	0.004*
ER (Positive vs. Negative)	1013	0.559 (0.351-0.891)	0.015*
HER2 (Positive vs. Negative)	704	1.477 (0.740-2.948)	0.269
Menopause status (Pre & peri vs. Post)	961	1.560 (0.871-2.794)	0.135
SEC61G expression (High vs. Low)	1062	2.348 (1.498-3.681)	<0.001*

Notes: The stages were graded according to AJCC7. Abbreviations: SEC61G, SEC61 translocon subunit gamma; AJCC7, American Joint Committee on Cancer 7th edition.

**Table 4:** Multivariate COX regression analysis for disease specific survival in the TCGA cohort.

Characteristics	Total(N)	HR(95% CI)	p-value
T stage (T2-3 vs. T1)	1059	0.689 (0.285-1.668)	0.409
N stage (N1-3 vs. N0)	1044	2.906 (1.508-5.601)	0.001*
M stage (M1 vs. M0)	903	3.598 (1.367-9.473)	0.010*
Pathologic stage (Stage II-IV vs. Stage I)	1041	2.249 (0.694-7.295)	0.177
Age (>60 vs. <=60)	1062	0.888 (0.493-1.598)	0.692
PR (Positive vs. Negative)	1010	0.749 (0.330-1.700)	0.490
ER (Positive vs. Negative)	1013	0.517 (0.217-1.232)	0.137
SEC61G expression (High vs. Low)	1062	2.162 (1.180-3.961)	0.013*

Notes: The stages were graded according to AJCC7. Abbreviations: SEC61G, SEC61 translocon subunit gamma; AJCC7, American Joint Committee on Cancer 7th edition.

**Table 5:** Connection between the expression level of SEC61G and the immune infiltration in the tumor microenvironment.

Immune cell	Spearman correlation	P value
aDC	0.134	<0.001*
B cells	0.024	0.419
CD8 T cells	-0.078	0.010*
Cytotoxic cells	0.025	0.413
DC	0.030	0.321
Eosinophils	-0.209	<0.001*
iDC	-0.118	<0.001*
Macrophages	0.057	0.057
Mast cells	-0.249	<0.001*
Neutrophils	0.011	0.705
NK CD56bright cells	-0.112	<0.001*
NK CD56dim cells	0.117	<0.001*
NK cells	-0.238	<0.001*
pDC	-0.133	<0.001*
T cells	-0.021	0.486
T helper cells	-0.176	<0.001*
Tcm	-0.291	<0.001*
Tem	-0.125	<0.001*
TFH	-0.047	0.116
Tgd	-0.056	0.065
Th1 cells	0.113	<0.001*
Th17 cells	-0.116	<0.001*
Th2 cells	0.276	<0.001*
TReg	0.125	<0.001*

Abbreviations: aDC: activated dendritic cell; DC: dendritic cell; iDC: immature dendritic cell; Macrophages; Mast cells; pDC: Plasmacytoid dendritic cell; Tcm: T central memory; Tem: T effector memory; Tfh: T follicular helper; Tgd: T gamma delta; Treg: T regulatoy cell.
