# Supplementary material for: SEC61G regulates breast cancer cell proliferation and metastasis by affecting the Epithelial-Mesenchymal Transition
Source: J Cancer. 2022 Jan 1;13(3):831–46. doi: 10.7150/jca.65879 (PMC8824897; doi:10.7150/jca.65879)
Supplement: Supplementary file 1 — Supplementary table. [file jcav13p0831s1.pdf]

Table S1: The basic information of IHC slices

Tissue type	ID	Age	Gender
Normal-1	3544	45	Female
Normal-2	3856	27	Female
Tumor -1	4193	43	Female
Tumor -2	4193	43	Female
